# Residual Medial Ankle Pain After the Delayed Union of a Lateral Malleolus Fracture: A Case Report

**DOI:** 10.7759/cureus.53112

**Published:** 2024-01-28

**Authors:** Akinobu Minagawa, Tadashi Kimura, Nori Yamashita, Mitsuru Saito, Makoto Kubota

**Affiliations:** 1 Department of Orthopaedic Surgery, Jikei University School of Medicine, Tokyo, JPN

**Keywords:** delayed union, ankle pain, ankle instability, osteochondral lesion, lateral malleolus fracture

## Abstract

A 17-year-old girl sprained her left ankle and was diagnosed with a lateral malleolar fracture. She was treated conservatively for six months but had medial ankle pain with activity. Imaging revealed an oblique lateral malleolar fracture, with posterolateral displacement and partial fusion of the bone fragments, and bone marrow edema on the medial articular surface of the talus and medial malleolus.

We diagnosed ankle instability due to delayed union with a displacement of the lateral malleolus, which caused an osteochondral lesion. We performed arthroscopic and open surgery eight months after the injury, reducted the lateral malleolus anatomically, and fixed it with a plate. Postoperatively, the pain improved rapidly, and the bone marrow edema had almost disappeared on an MRI.

In this case, we think rotational instability of the ankle mortise caused abnormal pressure and continuous stress on the medial malleolus after injury, which may have contributed to persistent medial ankle pain.

## Introduction

There is a case report showing good results with conservative treatment of undisplaced or minimally displaced lateral malleolus fractures in young patients [1]. However, conservative treatment of lateral malleolar fractures often results in malunion, which can lead to osteoarthritis as a result of changes in ankle motion [2, 3]. Therefore, only slight displacement is allowed, and, in general, displacements of the fibula, including shortening and lateral and posterior translation, are anatomically permitted by 2 mm [4-6].

Radiologically, posterior displacement is measured as the distance between bone fragments on lateral radiographs [7]. This report describes a case in which medial ankle pain persisted after the delayed union of a lateral malleolar fracture but resolved rapidly after anatomical reduction of the lateral malleolus.

## Case presentation

A 17-year-old girl twisted her left ankle when she landed on the floor during a basketball game. She was diagnosed with a distal fibula fracture and put in a cast for six weeks. After six months of rehabilitation, she was still unable to return to sport and was referred to our hospital with persistent pain from exercise and walking.

Initial examination revealed mild swelling of the ankle and tenderness of the medial malleolus. Pain was induced at the medial malleolus by applying external rotation stress to the ankle. The ankle range of motion was maintained at 15° in dorsiflexion and 45° in plantar flexion.

**Figure 1:**
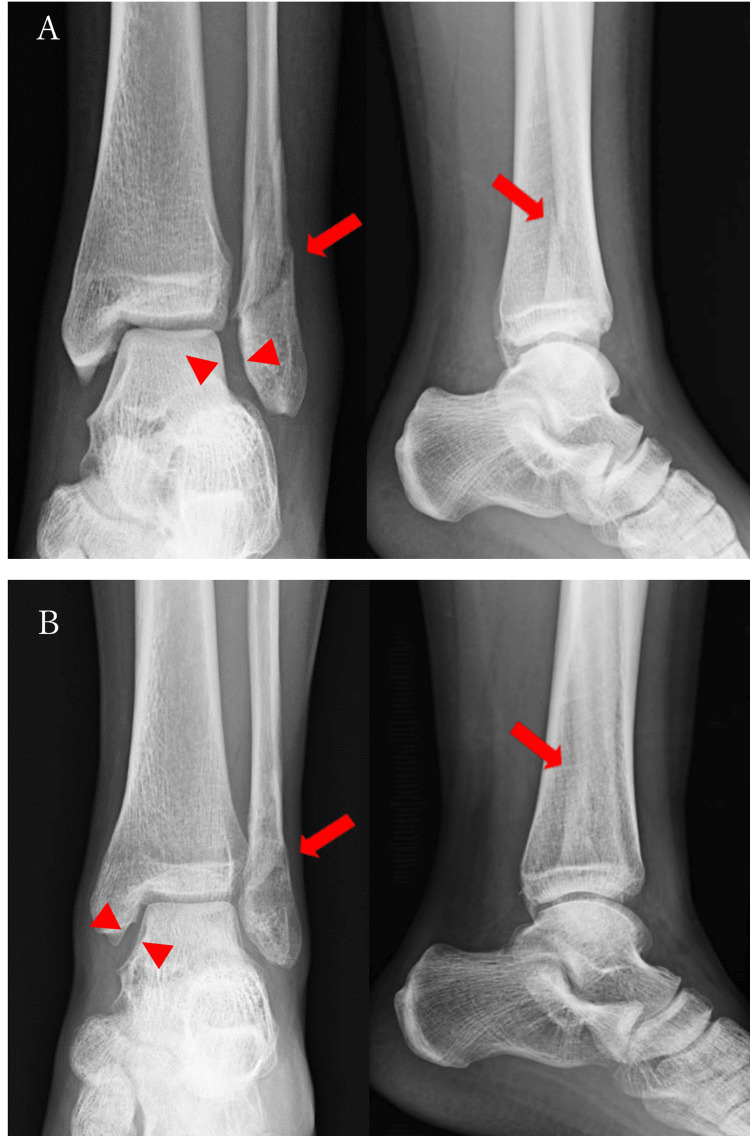
Radiographs of the patient (A) Plain X-ray frontal and lateral views obtained at the time of injury showed that the distal end of the fibula was obliquely fractured, displaced posterior-laterally, and 2.5 mm shorter (arrow), and the lateral joint space was dilated (arrowhead). (B) Plain X-ray frontal and lateral images obtained at the time of arrival showed mild enlargement of the medial joint space (arrowhead).

Plain radiographs at the time of injury showed a Weber type B oblique fracture of the distal fibula. This fracture ran from the level of the joint in the posterior-superior direction with approximately 2.5 mm of shortening, displacement posterolaterally, and widening of the lateral joint space (Figure 1A).

**Figure 2:**
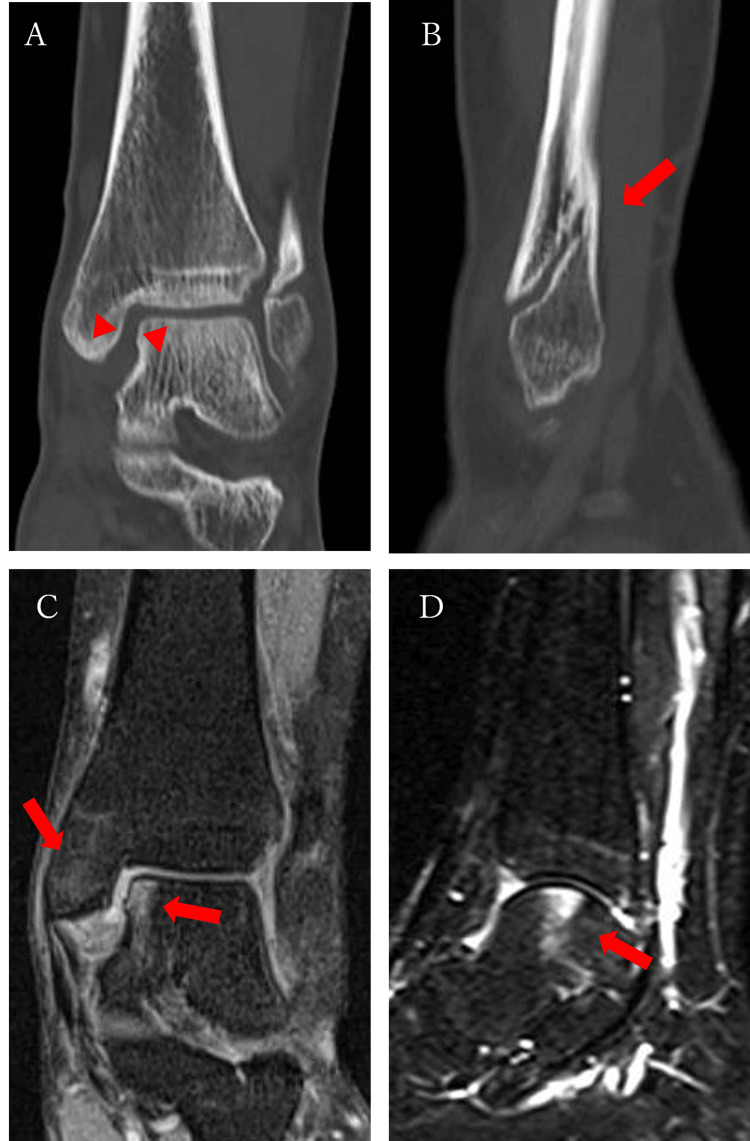
The patient's CT and MRI scans (A) A CT scan (coronal section) shows that the medial joint space has expanded (arrow). (B) A CT scan (sagittal section) shows that the peripheral bone fragments were displaced posterior-superiorly, and there was a gap left in the anterior part of the fracture, but the posterior bone was united (arrow). (C) An MRI proton density-weighted image (coronal section) shows a high signal which was observed on the medial articular surface of the talus and the medial malleolus. (D) An MRI short tau inversion recovery (STIR) image of the sagittal section shows a high signal which was observed on the medial articular surface of the talus and the medial malleolus.

The medial joint space was enlarged at the time of referral to our hospital (Figure 1B). Computed tomography (CT) scans showed posterior-superior displacement of the peripheral bone fragments, and although there was a gap in the anterior part of the fracture, the posterior part was fused (Figures 2A, 2B).

On the MRI, proton density-weighted and short tau inversion recovery (STIR) scans showed a high signal on the articular surface of the medial talus and medial malleolus but no significant damage to the deltoid ligament or anterior tibiofibular ligament (Figures 2C, 2D). In addition, there were marked signal changes in the medial malleolus, although six months had elapsed since the injury.

**Figure 3:**
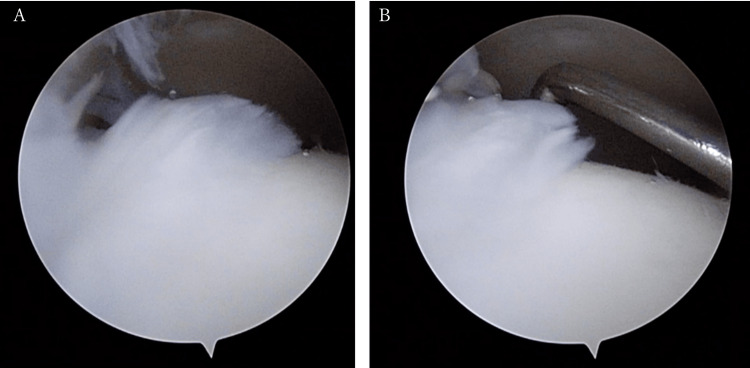
Findings of the ankle arthroscopy Panes (A) and (B) show detachment and roughening of the cartilage on the medial malleolar joint surface of the talus, and a part of them was trimmed.

The patient's pain was also unchanged, suggesting that there was ongoing stress on the medial malleolus after the injury. In addition, plain radiographs suggested that instability of the ankle joint was the cause of the ongoing stress. Based on the above findings, we diagnosed osteochondral disorder caused by instability of the ankle joint due to delayed union with displacement of the lateral malleolus. Eight months after the injury, we decided that surgery was necessary to stabilize the ankle joint and relieve the pain. Immediately before the surgery, when the ankle was stressed under fluoroscopy, the talus moved laterally, indicating instability of the ankle. First, ankle arthroscopy was performed with the patient in the semi-recumbent position. Detachment and fibrillation of the cartilage were observed on the medial malleolar surface of the talus, part of which was trimmed (Figure 3).

**Figure 4:**
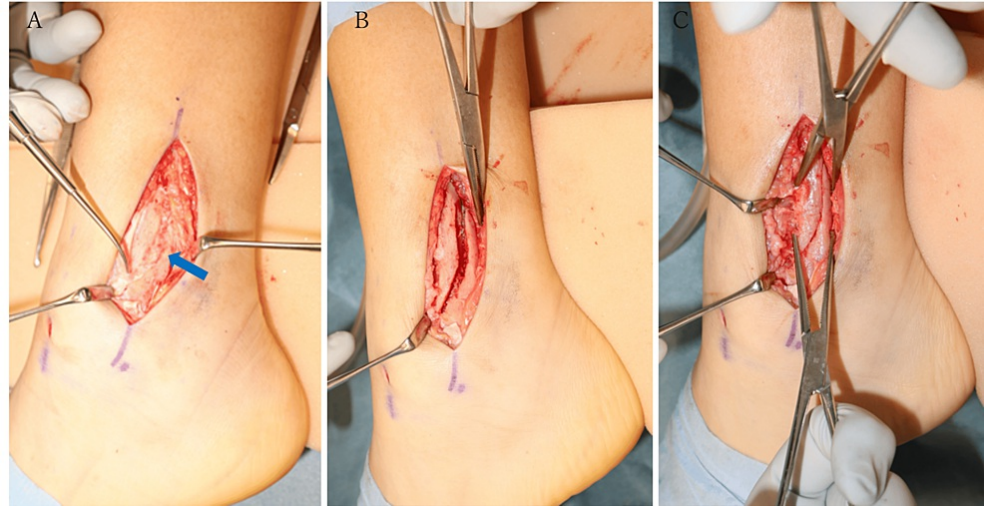
Intraoperative findings (A) The bone fragment was displaced posterior-superiorly and partially fused (arrow). (B) and (C) show that the fracture was freshened and reduced.

Next, a longitudinal skin incision was made at the posterior margin of the distal fibula to observe the delayed union. The distal bone fragments were displaced posterior-superiorly and partially fused, but the callus was still soft. After removing all the fibrous tissue and callus, we were able to see the original fracture and anatomically reduce it relatively easily (Figure 4).

**Figure 5:**
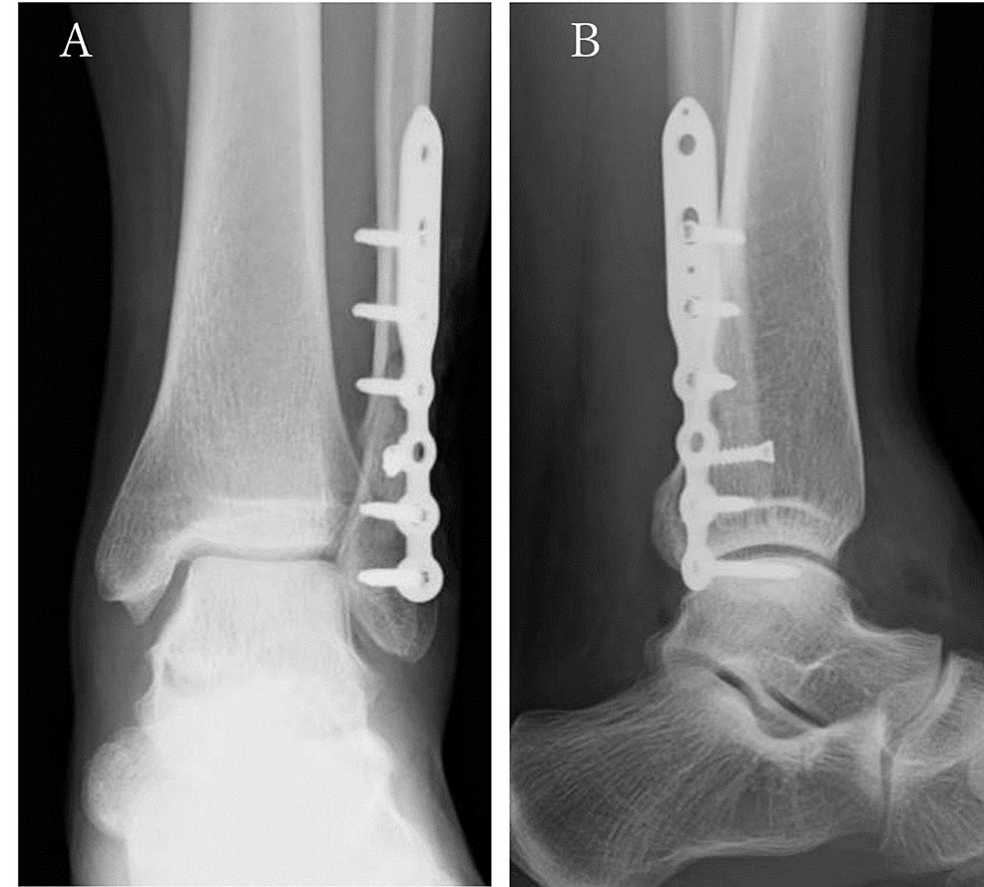
The patient's postoperative radiographs (A) Frontal view; (B) Lateral view

After posterolateral fixation with a lateral malleolus fracture plate, the instability disappeared (Figure 5).

Range of motion training was started two weeks after surgery, followed by partial weight bearing from four weeks and full weight bearing from seven weeks. Due to rapid improvement in pain and steady bone union, the patient was allowed to gradually return to exercise three months after surgery. The internal fixation material was removed 12 months after surgery.

Outcome

**Figure 6:**
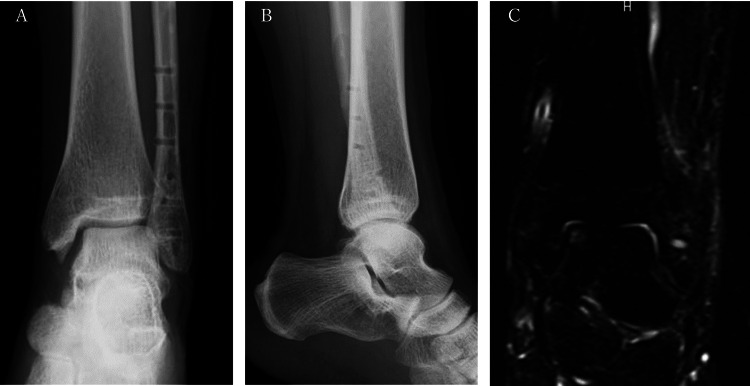
The patient's imaging results at the time of the final follow-up (A) Plain X-ray frontal view; (B) Plain X-ray lateral view; (C) MRI short tau inversion recovery (STIR) image coronal section shows that the bone marrow edema of the talus had almost disappeared.

At the time of the last follow-up (14 months after the first operation), the patient was pain-free and participating in sports activities without any problems. Plain radiographs indicated that the joint spaces between the medial and lateral sides of the talus were even, and an MRI showed that the bone marrow edema observed before surgery had largely resolved (Figure 6).

**Figure 7:**
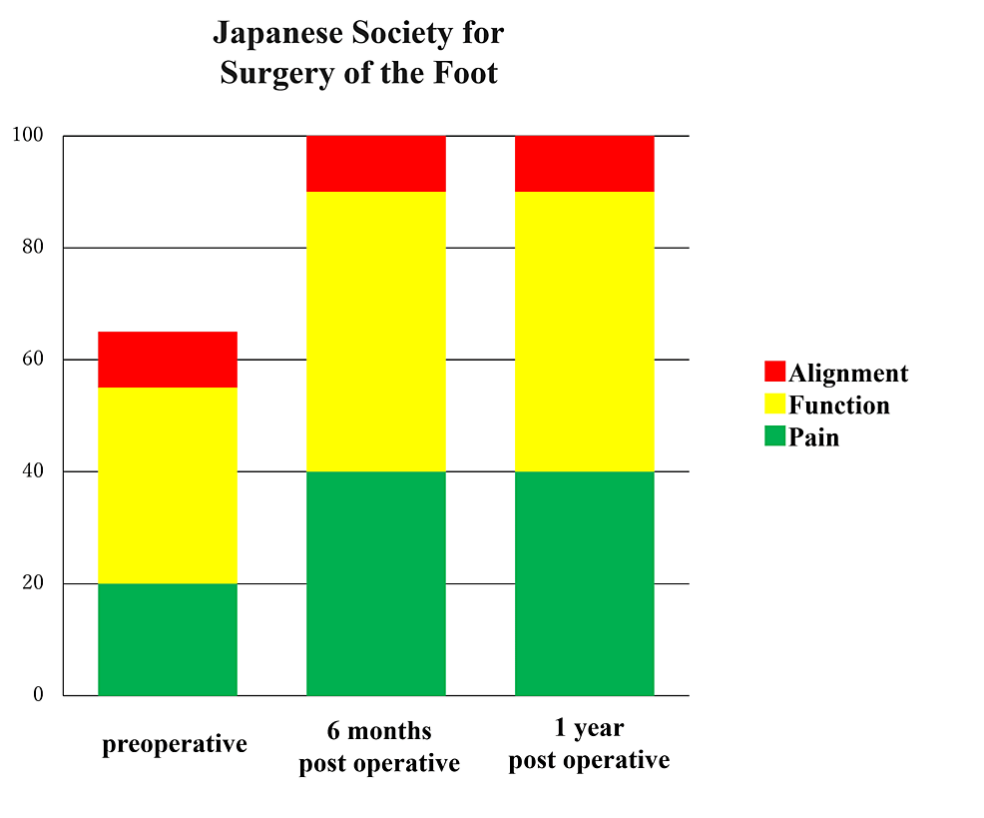
Japanese Society for Surgery of the Foot, Ankle/Hindfoot scale The score was 65 points before the operation but improved to 100 points six months after the surgery.

Her Japanese Society for Surgery of the Foot Ankle/Hindfoot scale [8, 9] increased from 65 points (with deductions for pain and function) preoperatively to 100 points at the six-month postoperative follow-up (Figure 7).

**Figure 8:**
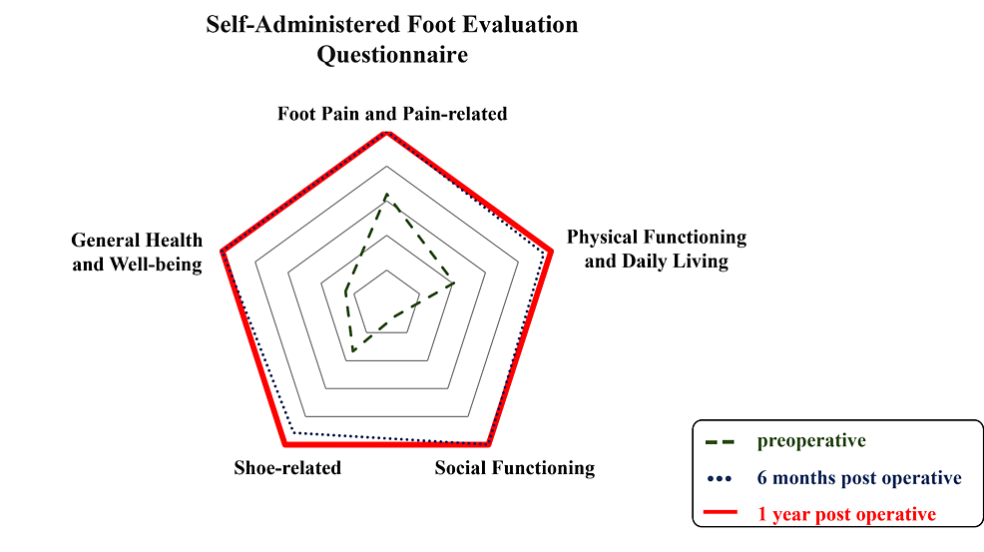
Self-Administered Foot Evaluation Questionnaire Prior to the operation, deductions were observed in social functioning, especially, but no deductions were observed in any of the categories one year after the operation.

Her preoperative Self-Administered Foot Evaluation Questionnaire scores [10, 11] were generally low, particularly for social functioning, but there was no deduction in any item at six and 12 months after surgery (Figure 8).

## Discussion

According to studies of ankle fractures conducted between 2016 and 2019 in the United States [12], the incidence of ankle fractures was 14.1 per 10,000 patient-years. The incidence of surgery for an ankle fracture was 3.3 per 10,000 patient-years and accounted for 23.4% of all fractures. The number of fractures increases during childhood and peaks at the age of 14. Then, it gradually decreases until the age of 29, after which it increases again with age. Among 15- to 19-year-olds, males had a higher incidence of fractures than females. However, after the age of 30, the incidence was higher in females in all age groups, and the gender difference widened with increasing age.

It has been reported that 39% of patients with ankle osteoarthritis have a history of ankle fractures [13] and that 14% develop osteoarthritis within two to six years after surgery [14]. Furthermore, it has been reported that 33%-67.3% of patients treated conservatively develop osteoarthritis [14]. In patients with ankle osteoarthritis, initial changes are observed 12-18 months after the injury, with progression to advanced osteoarthritis 10-20 years after the injury [15, 16]. It is well known that residual instability of the ankle mortise is a significant risk factor for ankle osteoarthritis. Ramsey and Hamilton [17] reported that the contact area between the tibia and talus decreased by 42% when the talus was displaced laterally by 1 mm. Burwell and Charnley [18] observed osteoarthritis of the ankle in cases where the medial and lateral malleoli were displaced ≥1 mm in the axial direction and in cases where the posterior malleolar fragment was displaced ≥2 mm proximally. These two reports have been widely cited. The most common residual deformity after conservative treatment is the shortening and rotation of the lateral malleolus [19], as seen in this case. In cadaveric studies, oblique fractures of the distal fibula at the level of the articular surface increased inversion and external rotation of the talus during the stance phase, and when the ankle joint was externally rotated under weight-bearing, the talus moved anterolaterally, inverted, and rotated externally [20, 21]. Therefore, the stability of the ankle mortise is considered to be greatly affected by the reduction of the fibula [22], and only slight dislocation, less than 2 mm, is permitted for lateral malleolar fractures [4-6].

In this case, the chief complaint was residual pain in the medial malleolus, and we cannot rule out the possibility that the bone marrow edema observed in the medial talus occurred at the time of injury. However, residual ankle instability after an ankle fracture may also damage the cartilage and aggravate the injury. It has been reported that when the fibula is shortened by >2 mm or externally rotated by >5°, the pressure increases on the medial side of the talar dome and the part from the mid-lateral to the posterolateral quadrant of the talar dome [4]. Although we could not identify the exact mechanism of injury, the lateral malleolar fracture was oblique, started at the level of the anterior joint, and was classified as an external rotation fracture according to the Haraguchi classification [23]. Given that the bone fragment was displaced and fused in the posterior-superior direction, it was considered that rotational instability remained in the external rotation direction, consistent with the mechanism of injury. This may have been associated with abnormal pressure on the medial articular surface of the ankle, excessive traction of the ligaments and joint capsule, and subsequent synovitis.

Hintermann et al. reported that 39.6% of ankle fractures had deltoid ligament damage [24], and another paper presented that MRI revealed deltoid ligament injury in 58.3% of ankle fractures [25]. Medial clear space (MCS) is considered less than 4 mm, which is normal [26]. In a report of stress testing performed after open reduction and internal fixation of ankle fractures, enlargement of the MCS was observed in patients with deltoid ligament injuries [27]. This suggests that damage to the deltoid ligament is involved in the enlargement of the MCS. In a study in which Weber's B fractures were divided into three groups: no repair of the deltoid ligament, repair of the superficial deltoid ligament, and repair of the deep deltoid ligament, no significant differences were observed in MCS and clinical scores [28]. However, there is also a report that the group in which the deltoid ligament was repaired in the ankle fracture showed improvement in MCS compared with the group in which the deltoid ligament was not repaired [29], and repair of the deltoid ligament is still controversial. In our case, we found a medial joint gap. However, an MRI showed no deltoid ligament injury, and the instability disappeared after fixation. We concluded that repair of the deltoid ligament was unnecessary. Furthermore, after anatomical reduction of the lateral malleolus, the pain on the medial side disappeared quickly, and the Japanese Society for Surgery of the Foot Ankle/Hindfoot scale and Self-Administered Foot Evaluation Questionnaire improved rapidly [8-11]. The MRI findings also indicated significant improvement 13 months after surgery. As with previous reports, this case highlights the need to maintain the ankle mortise in its anatomical form. We believe that the restoration of a stable ankle joint contributed greatly to the improvement in imaging findings and symptoms in this case.

There are some limitations in this case. One is that the mechanism of injury was not clear. In this case, the injury occurred during a basketball game, and some time had passed since the injury. The patient did not remember how she was injured. We finally considered the fracture to be an externally rotated fracture based on the imaging findings and performed surgery after confirming no obvious ligament injury. Although the results were good, it is important to have a good understanding of the mechanism of injury to identify sites of injury other than fractures, such as ligament injuries, and to select treatment methods. The second point is that we didn’t take stress radiographs, but we inferred the presence of instability from the differences in the plain radiographs from immediately after the injury to when the patient was referred to us and the disappearance of the talus displacement after surgery. The third point is that it cannot be determined whether the talar osteochondral disorder occurred at the time of the injury or not. We could not find any literature on conservative treatment of lateral malleolus fractures that compared MRI findings immediately after injury and during follow-up. However, it has been reported that MRI findings improve by six months postoperatively in most cases of osteochondral injury with an ankle fracture [30]. At the time of visiting our hospital, there was residual pain on the medial side of the ankle, and signal changes of the talus and medial malleolus were observed on the MRI performed six months post injury. It suggests that the medial malleolus was under continuous stress after the injury. Based on the improvement in symptoms and imaging findings after correction of ankle instability, ankle joint instability was considered to be the cause of the talar osteochondral lesion. The last point is that the follow-up period may have been short. The patient’s follow-up was completed 14 months after surgery, but there were no imaging findings or symptoms suggesting recurrence, and the patient was able to participate in sports without any problems. We instructed her to visit us if she had any problems, but four years later, she still has not contacted us.

## Conclusions

We encountered a case of residual pain in the medial ankle joint after the delayed union of a lateral malleolar fracture. The medial malleolus pain was considered to be related to ankle instability, and the symptoms improved rapidly after anatomical reduction of the lateral malleolus. This case highlights the importance of anatomical reduction of the lateral malleolus.
